# One-step removal of a migrated biliary plastic stent using a novel long thin dilating balloon catheter

**DOI:** 10.1055/a-2599-6714

**Published:** 2025-06-03

**Authors:** Noriyuki Hirakawa, Kenjiro Yamamoto, Takayoshi Tsuchiya, Ryosuke Tonozuka, Shuntaro Mukai, Takao Itoi

**Affiliations:** 1 13112 Gastroenterology and Hepatology, Tokyo Medical University, Tokyo, Japan

Biliary plastic stent migration in endoscopic retrograde cholangiopancreatography (ERCP)-related procedures is a complication that is occasionally encountered 1 . Techniques for removing migrated plastic stents have been reported 2 3 4 ; however, some cases are still challenging, even with the use of these techniques. Here, we report the one-step removal of a migrated plastic stent using a novel long dilating balloon.

The patient was a 59-year-old man who had undergone biliary drainage using a straight-type 7-Fr plastic stent for obstructive jaundice due to pancreatic head cancer. He was admitted with chief complaints of chills and right upper quadrant abdominal pain. An abdominal radiograph showed stent migration ( Fig. 1 ), so an emergent endoscopic procedure was performed. Endoscopic examination confirmed that the stent had become dislodged into the bile duct ( Fig. 2 ), so we therefore attempted stent removal. The bile duct was cannulated, and cholangiography showed a distal bile duct stricture. After endoscopic sphincterotomy had been performed, we tried but failed to grasp the distal end of the stent with grasping forceps under fluoroscopic guidance. A stone extraction balloon catheter was advanced to the bile duct, but could not be advanced beyond the stricture owing to the migrated stent.

**Fig. 1:**
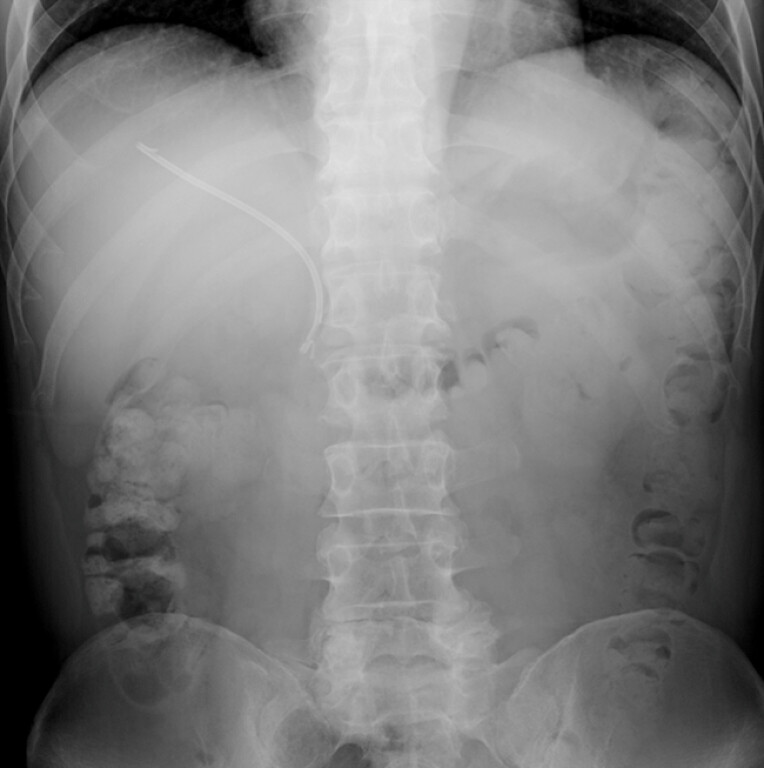
Abdominal radiograph showing the migrated plastic stent.

**Fig. 2:**
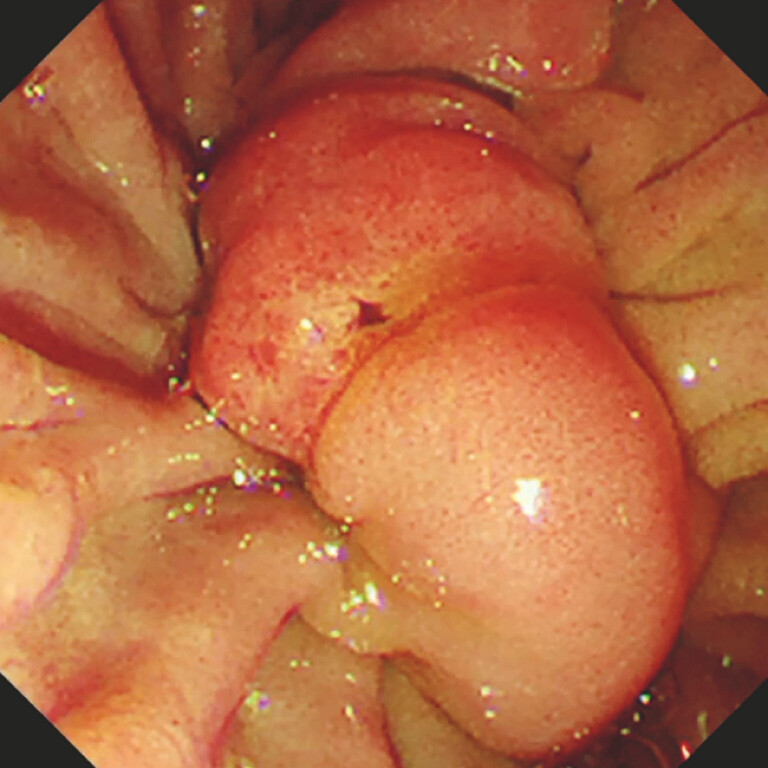
Endoscopic view showing the dislodged bile duct stent.

We next attempted to use a novel long thin dilating balloon catheter (3 mm × 6 cm; REN Biliary Dilation Catheter; Kaneka Medix, Osaka, Japan) ( Fig. 3 ) 5 . First, another guidewire (0.025-inch straight type) was advanced through the stent’s lumen under fluoroscopic guidance. The novel dilating balloon catheter was then inserted into the stent’s lumen ( Fig. 4 ) and inflated. The balloon catheter was next pulled gently and slowly backward, with the plastic stent finally being successfully removed through the scope without further difficulty ( Video 1 ). Removal was achieved as the inflated balloon expands into the flap hole, so that the balloon catheter becomes integrated with the plastic stent ( Fig. 5 ).

**Fig. 3:**
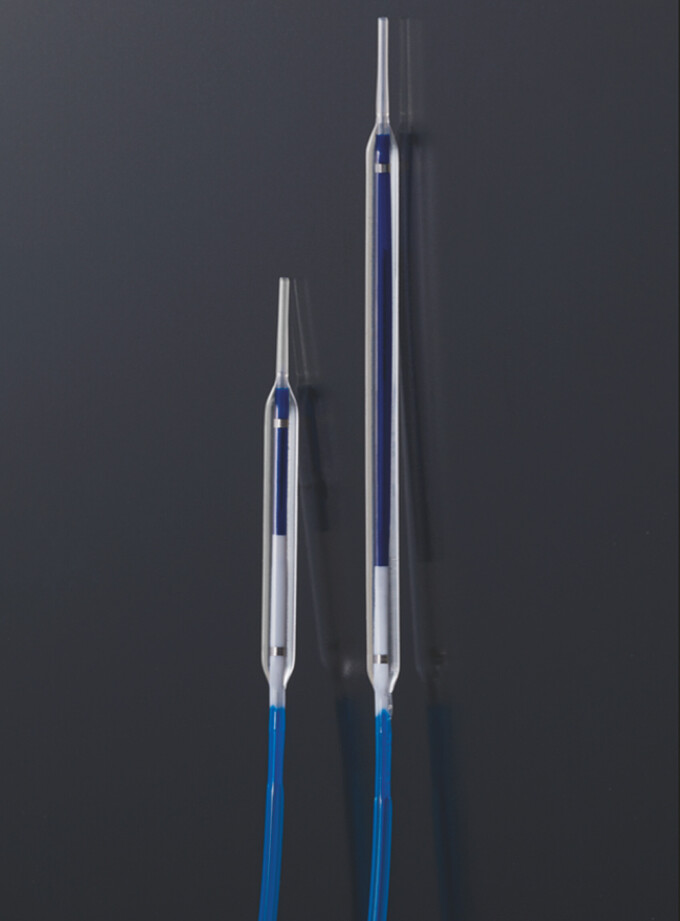
Photograph showing a conventional dilating balloon catheter (left) and the novel dilating balloon catheter (right).

**Fig. 4:**
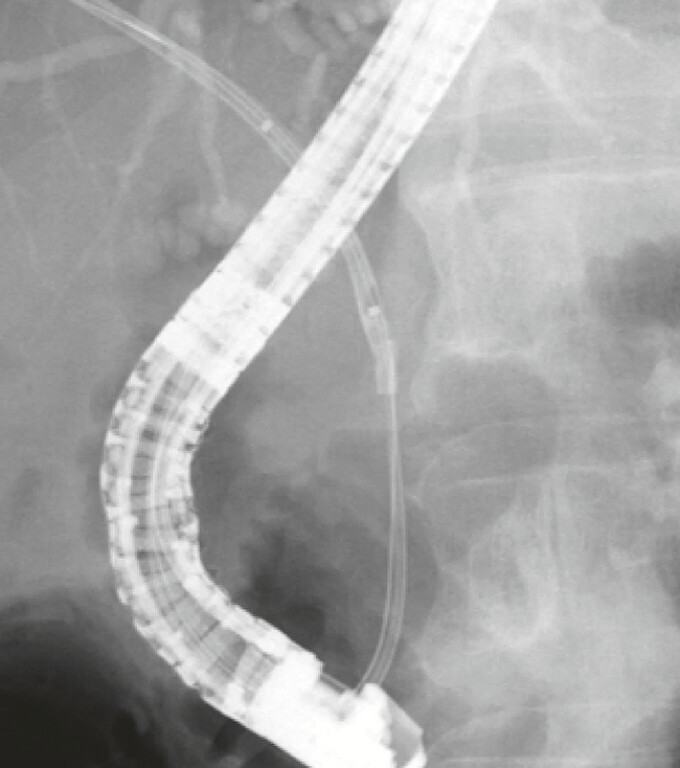
Fluoroscopic image showing the novel long thin dilating balloon (3 mm × 6 cm) inserted into the stent’s lumen.

One-step removal of a migrated biliary plastic stent using a novel long thin dilating balloon.Video 1

**Fig. 5:**
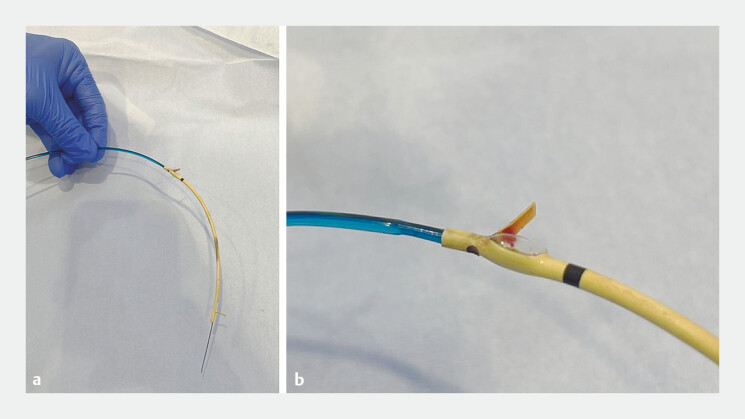
Photographs showing the inflated balloon expanding into the flap hole of the plastic stent, so that the balloon catheter has become integrated with the stent.

Endoscopy_UCTN_Code_CPL_1AK_2AD

## Notes

Endoscopy E-Videos https://eref.thieme.de/e-videos
*E-Videos* is an open access online section of the journal *Endoscopy* , reporting on interesting cases and new techniques in gastroenterological endoscopy. All papers include a high-quality video and are published with a Creative Commons CC-BY license. Endoscopy E-Videos qualify for HINARI discounts and waivers and eligibility is automatically checked during the submission process. We grant 100% waivers to articles whose corresponding authors are based in Group A countries and 50% waivers to those who are based in Group B countries as classified by Research4Life (see: https://www.research4life.org/access/eligibility/ ). This section has its own submission website at https://mc.manuscriptcentral.com/e-videos .
